# Four‐Year Follow‐up of [^18^F]Fluorodeoxyglucose Positron Emission Tomography–Based Parkinson's Disease–Related Pattern Expression in 20 Patients with Isolated Rapid Eye Movement Sleep Behavior Disorder Shows Prodromal Progression

**DOI:** 10.1002/mds.28260

**Published:** 2020-09-10

**Authors:** Rosalie V. Kogan, Annette Janzen, Sanne K. Meles, Elisabeth Sittig, Remco J. Renken, Vita Gurvits, Geert Mayer, Klaus L. Leenders, Wolfgang H. Oertel, and the REMPET Working Group, Jan Booij, Kathrin Reetz, Sebastiaan Overeem, Angelique Pijpers, Felix Bernhard, David Vállez García, Débora E. Peretti, Laura K. Teune, Fransje E. Reesink, Jelmer G. Kok

**Affiliations:** ^1^ Department of Nuclear Medicine and Molecular Imaging University of Groningen, University Medical Center Groningen Groningen the Netherlands; ^2^ Department of Neurology Philipps‐Universität Marburg Marburg Germany; ^3^ Department of Neurology University of Groningen, University Medical Center Groningen Groningen the Netherlands; ^4^ Department of Biomedical Sciences of Cells & Systems, Cognitive Neuroscience Center University of Groningen Groningen the Netherlands; ^5^ Institute for Neurogenomics Helmholtz Center for Health and Environment Munich Germany

**Keywords:** [^18^F]FDG‐PET, rapid eye movement sleep behavior disorder, Parkinson's disease–related pattern, neuroimaging, prodromal progression biomarker

## Abstract

**Background:**

Isolated rapid eye movement sleep behavior disorder is known to be prodromal for alpha‐synucleinopathies, such as Parkinson's disease (PD) and dementia with Lewy bodies. The [^18^F]fluorodeoxyglucose‐positron emission tomography (PET)–based PD‐related brain pattern can be used to monitor disease progression.

**Objective:**

We longitudinally investigated PD‐related brain pattern expression changes in 20 subjects with isolated rapid eye movement sleep behavior disorder to investigate whether this may be a suitable technique to study prodromal PD progression in these patients and to identify potential phenoconverters.

**Methods:**

Subjects underwent two [^18^F]fluorodeoxyglucose‐PET brain scans ~3.7 years apart, along with baseline and repeated motor, cognitive, and olfactory testing within roughly the same time frame.

**Results:**

At baseline, 8 of 20 (40%) subjects significantly expressed the PD‐related brain pattern (with *z* scores above the receiver operating characteristic–determined threshold). At follow‐up, six additional subjects exhibited significant PD‐related brain pattern expression (70% in total). PD‐related brain pattern expression increased in all subjects (*P* = 0.00008). Four subjects (20%), all with significant baseline PD‐related brain pattern expression, phenoconverted to clinical PD.

**Conclusions:**

Suprathreshold PD‐related brain pattern expression and greater score rate of change may signify greater shorter‐term risk for phenoconversion. Our results support the use of serial PD‐related brain pattern expression measurements as a prodromal PD progression biomarker in patients with isolated rapid eye movement sleep behavior disorder. © 2020 The Authors. *Movement Disorders* published by Wiley Periodicals LLC on behalf of International Parkinson and Movement Disorder Society

Isolated or idiopathic rapid eye movement sleep behavior disorder (iRBD) is known to be prodromal for alpha‐synucleinopathies, such as Parkinson's disease (PD), dementia with Lewy bodies (DLB), or more rarely, multiple system atrophy (MSA) in >80% of cases.^1^, ^2^, ^3^ As such, patients with iRBD are critical for the study of prodromal PD development and will likely be key to disease‐modifying drug trials.

However, this necessitates the availability of reliable biomarkers for the tracking and prediction of disease progression and phenoconversion to manifest alpha‐synucleinopathies. Equally important, these biomarkers must be able to identify those who will *not* phenoconvert.

Accordingly, this study focuses on the longitudinal change in expression of a characteristic pattern of [^18^F]fluorodeoxyglucose positron emission tomography ([^18^F]FDG‐PET)‐based abnormal cerebral glucose metabolism known as the “PD‐related pattern” (PDRP) in subjects with iRBD. The PDRP is defined by the Scaled Subprofile Model Principal Component Analysis (SSM/PCA) method,^4^, ^5^ and it has been widely used in the study of parkinsonian syndromes.^6^ Previous reports have demonstrated that PDRP expression precedes onset of motor symptoms by several years in prodromal patients,^7^, ^8^, ^9^ and that in manifest PD, pattern expression increases with disease progression^10^ and decreases with effective symptomatic treatment.^11^

Repeated longitudinal measurements of [^18^F]FDG‐PET‐based disease‐related metabolic brain pattern expression in patients with iRBD have not been performed before. We therefore studied 20 subjects with iRBD with glucose metabolic brain imaging, as well as motor, cognitive, and olfactory testing two times approximately 4 years apart. Our primary purpose was to investigate whether serial PDRP expression measurements may be a suitable technique to study prodromal PD progression in patients with iRBD.

## Methods

### Study Design and Participants

This prospective, two‐part longitudinal pilot study took place at the University Medical Center of Groningen in Groningen, the Netherlands, and at the Philipps‐Universität Marburg in Marburg, Germany. Study protocols for the baseline and follow‐up investigations were approved by the institutional review boards of both institutions, and voluntary informed consent was obtained from each subject at baseline and follow‐up after verbal and written explanation of the study, in accordance with the Declaration of Helsinki.

Twenty subjects with iRBD (3 Dutch and 17 German) were evaluated with baseline and follow‐up [^18^F]FDG‐PET imaging, as well as motor, cognitive, and olfactory testing (see Table 1 for demographics). At baseline, subjects with iRBD with a clinical diagnosis of parkinsonism, dementia, or history of psychotropic medication use before or during iRBD onset were excluded.

**TABLE 1:** Results are ordered from lowest to highest follow‐up PDRP expression *z* score

Subject no.	Gender	Age (yr)	Age of iRBD onset (yr)	Duration of iRBD (yr)	Baseline PDRP *z* score	Follow‐up PDRP *z* score category	Follow‐up PDRP *z* score (change from baseline)	PDRP change per year	Sniffin' Sticks Identification Test (change from baseline)	UPDRS‐III (change from baseline)	MoCA (change from baseline)
1	M	60.4	52.8	7.6	−1.56	<1.98	−0.77 (+0.80)	0.31	10 (−2)^a^	0 (−1)	27 (−1)
2	M	61.2	55.0	6.2	−2.19		−0.72 (+1.47)	0.40	13 (+4)	3 (−3)	29 (+2)
3	M	61.4	52.4	9.0	−0.47		0.70 (+1.18)	0.30	12 (−1)	1 (−3)	28 (−2)
4	M	58.0	48.0	10.0	−0.08		0.93 (+1.01)	0.25	13 (+2)	7 (+3)^a^ ^,^ ^b^	27 (+1)
5	M	67.1	54.1	13.0	−0.59		1.40 (+1.98)	0.50	7 (−2)^a^	2 (0)	29 (0)
6	M	70.1	42.1	28.0	1.42		1.77 (+0.35)	0.12	8 (+2)^a^	0 (0)	28 (0)
7	F	72.5	62.3	10.3	−0.85	≥1.98	2.07 (+2.92)^a^ ^,^ ^c^	0.68	14 (0)	2 (0)	30 (+7)
8	M	69.9	63.9	6.0	1.00		2.44 (+1.44)^a^ ^,^ ^c^	0.48	12 (+2)	4 (+2)	27 (0)
9	M	67.5	62.5	5.0	−0.71		2.60 (+3.30)^a^ ^,^ ^c^	1.10	6 (−2)^a^	4 (+2)	29 (+3)
10	M	69.9	60.4	9.4	1.23		2.74 (+1.52)^a^ ^,^ ^c^	0.44	5 (−2)^a^	4 (+1)	26 (−1)
11	M	64.1	57.5	6.6	0.35		3.02 (+2.67)^a^ ^,^ ^c^	1.04	5 (−1)^a^	0 (0)	30 (+3)
12	M	69.1	59.4	9.7	2.76^a^		3.72 (+0.97)^a^	0.26	2 (−3)^a^	2 (−4)	28 (+1)
13	M	68.1	50.0	18.0	0.60		4.16 (+3.56)^a^ ^,^ ^c^	0.88	12 (0)	3 (−1)	27 (−1)
14	F	74.7	67.1	7.6	2.18^a^		4.41 (+2.23)^a^	0.48	3 (−2)^a^	3 (−1)	30 (+2)
15	M	65.1	60.1	5.0	3.13^a^		4.46 (+1.33)^a^	0.35	4 (−4)^a^	1 (+1)	26 (−2)
16*	**M**	**66**.**6**	**61**.**6**	**5.0**	**2.10** ^a^		**5**.**25 (+3**.**15)** ^a^	**0**.**79**	**11 (+3)**	**7 (+7)** ^a^	**27 (−2)**
17	M	70.6	64.6	6.0	4.32^a^		6.19 (+1.87)^a^	0.48	0 (0)^a^	3 (−2)	29 (+7)
18*	**M**	**69**.**9**	**53**.**9**	**16.0**	**2.64** ^a^		**6**.**30 (+3**.**66)** ^a^	**0**.**94**	**6 (−2)** ^a^	**9 (+7)** ^a^	**28 (+2)**
19*	**M**	**67**.**2**	**59**.**2**	**8.0**	**4.68** ^a^		**7**.**67 (+2**.**99)** ^a^	**0**.**74**	**0 (0)** ^a^	**7 (+6)** ^a^	**28 (0)**
20*	**M**	**53**.**9**	**45**.**9**	**8.0**	**4.73** ^a^		**9**.**11 (+4**.**37)** ^a^	**1**.**08**	**2 (0)** ^a^	**15 (+14)** ^a^	**25 (+1)** ^a^
Mean ± SD		66.37 ± 5.17	56.64 ± 6.70	9.72 ± 5.55	1.23 ± 2.05		3.37 ± 2.63 (+2.14 ± 1.12)	0.58 ± 0.30	7.3 ± 4.6 (−0.4 ± 2.1)	3.7 ± 3.2 (+1.3 ± 3.9)	27.9 ± 1.4 (+1.0 ± 2.6)

Underlines denote subjects scanned on the Siemens Biograph mCT40 PET/CT scanner (the rest were scanned on the mCT64 system). Boldface and asterisk by subject number denote the four phenoconverted subjects. Given age and duration of iRBD are for the time of follow‐up [^18^F]fluorodeoxyglucose positron emission tomography ([^18^F]FDG‐PET) scan.

^a^ Denotes pathological results.

^b^ Subject 4 has an artificially elevated UPDRS motor score because of unrelated back problems.

^c^ Subjects 7–11 and 13 had subthreshold PDRP expression at baseline but suprathreshold scores at follow‐up [^18^F]FDG‐PET imaging.

Abbreviations: PDRP, Parkinson's disease–related pattern; iRBD, isolated or idiopathic rapid eye movement sleep behavior disorder; UPDRS‐III, Unified Parkinson's Disease Rating Scale, Part III; MoCA, Montreal Cognitive Assessment; M, male; F, female; SD, standard deviation.

In addition, 16 age‐ and gender‐matched healthy control subjects (HCs) (13 male/3 female, age 63.1 ± 6.7 years) underwent baseline [^18^F]FDG‐PET imaging to *z*‐transform the iRBD PDRP scores.

Exclusion criteria for all subjects at baseline included a history of (other) neurological diseases, diabetes mellitus, hyperthyroidism or hypothyroidism, stroke, significant head trauma, or other relevant comorbidities.

At both centers, phenoconversion to PD or DLB was determined by the neurologist performing the motor examination, according to the UK Parkinson's Disease Society Brain Bank diagnostic criteria or the DLB Consortium consensus criteria.^12^, ^13^ In addition, at Philipps‐Universität Marburg, a neurologist confirmed the presence of PD/DLB twice, 3 months apart, in phenoconverted German subjects.

### [^18^F]FDG‐PET

Twenty RBD Screening Questionnaire–screened and video polysomnographically confirmed iRBD patients underwent baseline and follow‐up [^18^F]FDG‐PET imaging an average of 3.7 ± 0.6 years apart. Sixteen HCs underwent baseline [^18^F]FDG‐PET imaging as well. All baseline and follow‐up scans were performed on a Siemens Biograph mCT64 or mCT40 PET/CT camera (Siemens, Munich, Germany) at the University Medical Center of Groningen. Images were reconstructed with OSEM3D (3 iterations, 21 subsets), time‐of‐flight, point‐spread‐function, Gaussian 8‐mm full‐width‐at‐half‐maximum spatial filter, and matrix size 256 (corresponding to a voxel size of 2 mm × 3.18 mm × 3.18 mm).

Central nervous system depressants and any iRBD‐related medications (ie, melatonin or clonazepam) were discontinued in all HCs and subjects with iRBD for at least 24 hours before baseline and follow‐up imaging.

All iRBD and HC images were spatially normalized to an [^18^F]FDG‐PET template in Montreal Neurological Institute brain space^14^ using SPM12 software (Wellcome Centre for Human Neuroimaging, London, UK) implemented in MATLAB (version R2019a; MathWorks, Natick, MA, USA).

A PDRP based on 16 HCs and 14 patients with PD was defined with the SSM/PCA^4^ method using data prepared to the same specifications as the earlier iRBD and HC data (see Supporting Information Fig. S1) (notably, these 16 HCs are a separate cohort from the one described earlier).

PDRP expression in subjects with iRBD and HCs was calculated using in‐house code. PDRP subject scores were *z*‐transformed to HCs such that the mean HC PDRP *z* score was 0, with a standard deviation of ±1. A receiver operating characteristic (ROC) analysis was performed to best differentiate between HCs and patients with PD, which determined a *z* score cutoff of 1.98 (specificity 100%, sensitivity 85.7%; high specificity will be an important consideration for drug trials to take into account to minimize misclassification of nonphenoconverters).

A Hoffman 3D Brain Phantom allowed for linear correction of raw PDRP score offsets between the mCT40 and mCT64 scanners (see Supporting Information).

### Motor, Cognitive, and Olfactory Assessment

All 20 subjects with iRBD were additionally assessed using the Unified Parkinson's Disease Rating Scale, Part III (UPDRS‐III; motor examination)^15^, ^16^ the Montreal Cognitive Assessment (MoCA),^17^ and the Sniffin' Sticks 16‐item olfactory odor identification test^18^ at baseline and follow‐up. A 5‐point change in the UPDRS‐III was considered to be clinically significant.^19^ MoCA scores ≤25 out of 30, and Sniffin' Sticks identification score ≤10 out of 16 were considered to be pathological.^17^, ^18^

### Statistical Analysis

Variables were tested for normality of distribution with the Shapiro‐Wilk test, and PDRP *z* scores and age were subsequently considered to be distributed parametrically.^7^

The rest of the variables (UPDRS‐III, MoCA, and olfactory scores; years duration of iRBD; and variables' rates of change) were considered to be nonparametrically distributed. Correlations between these and PDRP *z* scores were compared with a two‐sided Spearman rank correlation coefficient.

A one‐sample Wilcoxon signed rank test was used to examine PDRP *z* score change per year, UPDRS‐III score change per year, MoCA score change per year, and olfactory score change per year. A Spearman rank correlation coefficient was used to compare PDRP *z* score change per year with UPDRS‐III score change per year, MoCA score change per year, and olfactory score change per year.

These analyses were not corrected for multiple comparisons. Correlations were considered to be significant at *P* < 0.05 (uncorrected). All analyses were performed using SPSS v.24 (SPSS Inc., Chicago, IL).

Due to small subgroup sizes, we did not run statistical analyses based on phenoconversion status. Instead, these results are described qualitatively.

This study is registered with the Netherlands Trial Register, number NL8057.

## Results

Between 2014 and 2015, 20 patients with iRBD underwent baseline [^18^F]FDG‐PET imaging, as well as motor, cognitive, and olfactory testing (see Table 1).

**FIG 1:**
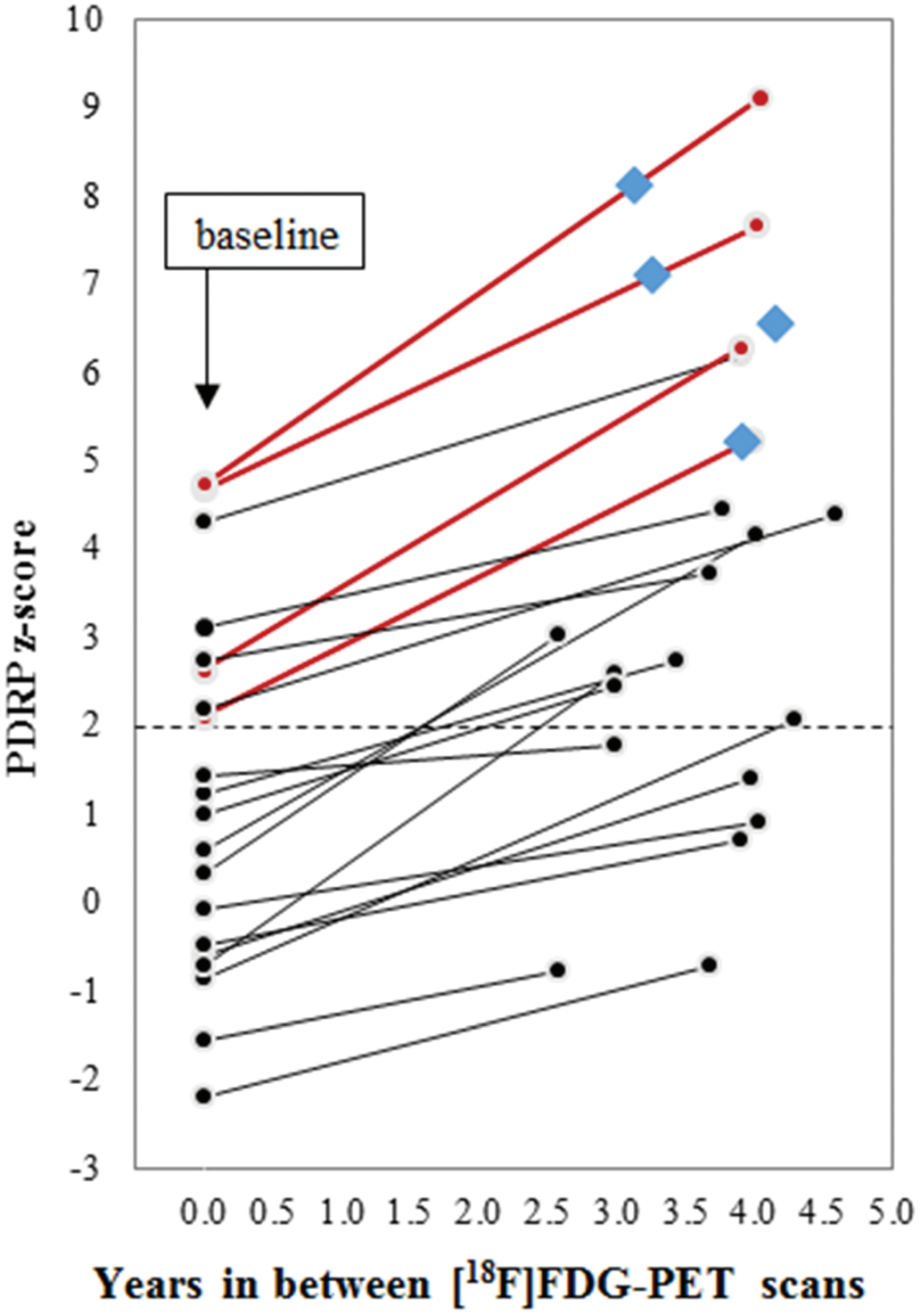
PDRP expression changes between baseline and follow‐up [^18^F]FDG‐PET imaging. Burgundy lines denote phenoconverted subjects; diamonds denote point of clinical phenoconversion.

At baseline, 8 of 20 (40%) subjects with iRBD expressed a PDRP *z* score above the ROC‐determined threshold of 1.98. At follow‐up, six additional subjects with iRBD exhibited suprathreshold PDRP *z* scores, for a total of 14 of 20 (70%). PDRP expression increased among all 20 (100%) subjects between baseline and follow‐up (see Fig. 1). At the group level, PDRP expression was significantly higher at follow‐up than at baseline (Wilcoxon *P* = 0.00008). The average absolute *z* score increase was by 2.1 ± 1.1 points, with an average *z* score increase of 0.6 ± 0.3 point per year.

UPDRS‐III, MoCA, and olfactory scores did not change significantly between baseline and follow‐up testing (Wilcoxon *P* < 0.33, 0.48, and 0.26, respectively). However, olfactory scores correlated significantly with PDRP *z* scores at baseline and follow‐up (Spearman *P* < 0.0005 and *P* = 0.001, respectively; see Supporting Information Fig. S2), although olfactory score change per year did not correlate to PDRP scores or PDRP change per year. UPDRS motor scores correlated with PDRP *z* scores at follow‐up, but not at baseline (Spearman *P* = 0.017 vs. 0.099). In addition, UPDRS‐III score change per year did correlate significantly with PDRP *z* score change per year (Spearman *P* = 0.024; see Supporting Information Fig. S3). MoCA scores and MoCA change per year did not correlate to PDRP *z* scores or PDRP change per year. In fact, all follow‐up MoCA scores were within the normal range except for one (25), which was from a phenoconverted subject with the highest PDRP *z* score of the entire iRBD cohort (9.11).

No significant correlation was found between age of iRBD onset, age or duration of iRBD at time of follow‐up, and any other variable tested in this study.

All four phenoconverters had suprathreshold baseline and follow‐up PDRP *z* scores, as well as greater PDRP score rate of change (see Fig. 1).

## Discussion

For the first time, this follow‐up study of serial [^18^F]FDG‐PET imaging demonstrates that the expression of abnormal cerebral metabolism in patients with iRBD increases over time. Our results support the use of serial [^18^F]FDG‐PET imaging and PDRP expression measurements as a prodromal disease progression biomarker in patients with iRBD. Previous cross‐sectional findings demonstrated that abnormal metabolic expression in iRBD can begin years before motor manifestations of alpha‐synucleinopathies.^7^

Motor, cognitive, and olfactory scores did not change significantly between baseline and follow‐up. This may be attributable to bias because of the test‐retest effect (with the MoCA) or guessing (in the case of the olfaction test).^20^ Olfaction has previously been reported not to be a disease progression biomarker in iRBD.^21^ In contrast, [^18^F]FDG‐PET showed consistent, significant changes between baseline and follow‐up imaging, which corresponded to changes in motor function. Based on the four phenoconverted subjects, we infer that subjects with suprathreshold absolute PDRP expressions and higher PDRP rates of change may be at shorter‐term risk for phenoconversion to clinical alpha‐synucleinopathy. This study underscores the importance of repeated PDRP measurements for its implementation as a disease progression biomarker.

One of the strengths of this study includes its longitudinal nature. Some of the limitations of this study include small sample size and lack of repeat measurements of the HC cohort for comparison. Investigation of greater numbers of subjects with iRBD will be necessary to confirm or modify the conclusions of this study. In addition, a separate investigation into expressions of the DLB‐ or MSA‐related patterns in this cohort was beyond the scope of this report.^6^, ^22^

Further longitudinal studies should also examine the relationship between [^18^F]FDG‐PET and other biomarkers, such as dopamine transporter imaging with [^123^I]*N*‐ω‐fluoropropyl‐2β‐carbomethoxy‐3β‐(4‐iodophenyl)nortropane single‐photon emission computed tomography ([^123^I]FP‐CIT‐SPECT).^23^, ^24^ The latter is a well‐studied potential prodromal progression biomarker in alpha‐synucleinopathies. However, serial [^18^F]FDG‐PET imaging may have an advantage, because [^18^F]FDG‐PET allows for quantitative analysis of changes in glucose uptake in all areas of the brain at once, whereas [^123^I]FP‐CIT‐SPECT is predominately used to visualize the dopaminergic nigrostriatal tract. [^18^F]FDG‐PET also likely has greater potential to identify which specific parkinsonian disorder will develop in a patient with iRBD.^6^, ^22^ In addition, it is known that some patients with DLB may have initially negative [^123^I]FP‐CIT‐SPECT scans.^25^

One hundred subjects with iRBD are currently being recruited for ongoing multicenter, multinational research within the scope of this project to validate our findings.

## Author Roles

Klaus L. Leenders, Wolfgang H. Oertel, Geert Mayer, Jan Booij, Kathrin Reetz, Sebastiaan Overeem, Angelique Pijpers, and Rosalie V. Kogan assisted in the study protocol and design. Klaus L. Leenders and Wolfgang H. Oertel secured the funding for this project. Annette Janzen, Felix Bernhard, Elisabeth Sittig, Sebastiaan Overeem, and Angelique Pijpers recruited patients. Sanne K. Meles, Laura K. Teune, Fransje E. Reesink, and Jelmer G. Kok recruited HCs. Elisabeth Sittig organized and accompanied German patients on visits to Groningen for [^18^F]FDG‐PET imaging. Rosalie V. Kogan organized all logistics and imaging appointments for patients in Groningen. Annette Janzen performed all of the neuroclinical assessments with German patients in Marburg, while Rosalie V. Kogan, Vita Gurvits, and Klaus L. Leenders performed the neuroclinical assessments with Dutch patients in Groningen. Rosalie V. Kogan performed all of the statistical analyses with help from Remco J. Renken and put together all of the figures and table with the support of Annette Janzen, with the exception of Supporting Information Fig. S1, which Sanne K. Meles made; Fig. 1 was also made with input from Sanne K. Meles. Remco J. Renken, David Vállez García, and Débora E. Peretti wrote the in‐house code used. Rosalie V. Kogan drafted the manuscript. All authors contributed commentary for revising the manuscript.

## Financial Disclosures

This study was funded by Dutch “Stichting ParkinsonFonds” and the German “ParkinsonFonds Deutschland. The funders of this study did not have any role in designing this study, collecting, analyzing, or interpreting the data, or in writing this article. The corresponding author had access to and responsibility for all of the relevant data that were submitted for publication. The authors report no conflicts of interest. W.H.O. is a Hertie Senior Research Professor (Charitable Hertie Foundation, Germany).

## Appendix

REMPET Working Group list of authors: Jan Booij, MD, PhD, Department of Radiology and Nuclear Medicine, Amsterdam University Medical Centers, location Academic Medical Center, Amsterdam, the Netherlands; Kathrin Reetz, MD, Department of Neurology and JARA‐BRAIN Institute Molecular Neuroscience and Neuroimaging, RWTH Aachen University, Aachen, Germany; Sebastiaan Overeem, MD, PhD, Kempenhaeghe Sleep Medicine Center, Heeze, the Netherlands; Angelique Pijpers, MD, PhD, Kempenhaeghe Sleep Medicine Center, Heeze, the Netherlands; Felix Bernhard, MD, Department of Neurology, Philipps‐Universität Marburg, Marburg, Germany; David Vállez García, PhD, Department of Nuclear Medicine and Molecular Imaging, University of Groningen, University Medical Center Groningen, the Netherlands; Débora E. Peretti, Department of Nuclear Medicine and Molecular Imaging, University of Groningen, University Medical Center Groningen, the Netherlands; Laura K. Teune, MD, PhD, Department of Neurology, University of Groningen, University Medical Center Groningen, the Netherlands, and Department of Neurology, Wilhemina Hospital Assen, the Netherlands; Fransje E. Reesink, MD, PhD, Department of Neurology, University of Groningen, University Medical Center Groningen, the Netherlands; and Jelmer G. Kok, MD, PhD, Department of Neurology, University of Groningen, University Medical Center Groningen, the Netherlands.

## Supporting information


**Appendix S1**. Supporting Information

## References

[1] Schenck CH , Boeve BF , Mahowald MW . Delayed emergence of a parkinsonian disorder or dementia in 81% of older men initially diagnosed with idiopathic rapid eye movement sleep behavior disorder: a 16‐year update on a previously reported series. Sleep Med 2013;14(8):744–748. 23347909 10.1016/j.sleep.2012.10.009

[2] Postuma RB , Iranzo A , Hu M , Hogl B , Boeve BF , Manni R , et al. Risk and predictors of dementia and parkinsonism in idiopathic REM sleep behaviour disorder: a multicentre study. Brain 2019;142(3):744–759. 30789229 10.1093/brain/awz030 PMC6391615

[3] Iranzo A , Fernandez‐Arcos A , Tolosa E , Serradell M , Molinuevo JL , Valldeoriola F , et al. Neurodegenerative disorder risk in idiopathic REM sleep behavior disorder: study in 174 patients. PLoS One 2014;9(2):e89741. 24587002 10.1371/journal.pone.0089741 PMC3935943

[4] Spetsieris P , Ma Y , Peng S , Ko JH , Dhawan V , Tang CC , et al. Identification of disease‐related spatial covariance patterns using neuroimaging data. J Vis Exp 2013;76:e50319. 10.3791/50319 PMC3728991 23851955

[5] Eidelberg D . Metabolic brain networks in neurodegenerative disorders: a functional imaging approach. Trends Neurosci 2009;32(10):548–557. 19765835 10.1016/j.tins.2009.06.003 PMC2782537

[6] Teune LK , Renken RJ , Mudali D , De Jong BM , Dierckx RA , Roerdink JB , et al. Validation of parkinsonian disease‐related metabolic brain patterns. Mov Disord 2013;28(4):547–551. 23483593 10.1002/mds.25361

[7] Meles SK , Vadasz D , Renken RJ , Sittig‐Wiegand E , Mayer G , Depboylu C , et al. FDG PET, dopamine transporter SPECT, and olfaction: combining biomarkers in REM sleep behavior disorder. Mov Disord 2017;32(10):1482–1486. 28734065 10.1002/mds.27094 PMC5655750

[8] Tang CC , Poston KL , Dhawan V , Eidelberg D . Abnormalities in metabolic network activity precede the onset of motor symptoms in Parkinson's disease. J Neurosci 2010;30(3):1049–1056. 20089913 10.1523/JNEUROSCI.4188-09.2010 PMC2866050

[9] Holtbernd F , Gagnon JF , Postuma RB , Ma Y , Tang CC , Feigin A , et al. Abnormal metabolic network activity in REM sleep behavior disorder. Neurology 2014;82(7):620–627. 24453082 10.1212/WNL.0000000000000130 PMC3963420

[10] Huang C , Tang C , Feigin A , Lesser M , Ma Y , Pourfar M , et al. Changes in network activity with the progression of Parkinson's disease. Brain 2007;130(pt 7):1834–1846. 17470495 10.1093/brain/awm086 PMC4454378

[11] Rodriguez‐Rojas R , Pineda‐Pardo JA , Martinez‐Fernandez R , Kogan RV , Sanchez‐Catasus CA , Del Alamo M , et al. Functional impact of subthalamotomy by magnetic resonance‐guided focused ultrasound in Parkinson's disease: a hybrid PET/MR study of resting‐state brain metabolism. Eur J Nucl Med Mol Imaging 2020;47(2):425–436. 31705173 10.1007/s00259-019-04497-z

[12] Gibb WR , Lees AJ . The relevance of the Lewy body to the pathogenesis of idiopathic Parkinson's disease. J Neurol Neurosurg Psychiatry 1988;51(6):745–752. 2841426 10.1136/jnnp.51.6.745 PMC1033142

[13] McKeith IG , Boeve BF , Dickson DW , Halliday G , Taylor JP , Weintraub D , et al. Diagnosis and management of dementia with Lewy bodies: fourth consensus report of the DLB Consortium. Neurology 2017;89(1):88–100. 28592453 10.1212/WNL.0000000000004058 PMC5496518

[14] Della Rosa PA , Cerami C , Gallivanone F , Prestia A , Caroli A , Castiglioni I , et al. A standardized [18F]‐FDG‐PET template for spatial normalization in statistical parametric mapping of dementia. Neuroinformatics 2014;12(4):575–593. 24952892 10.1007/s12021-014-9235-4

[15] Movement Disorder Society Task Force on Rating Scales for Parkinson's Disease . The Unified Parkinson's Disease Rating Scale (UPDRS): status and recommendations. Mov Disord 2003;18(7):738–750. 12815652 10.1002/mds.10473

[16] Goetz CG , Tilley BC , Shaftman SR , Stebbins GT , Fahn S , Martinez‐Martin P , et al. Movement Disorder Society‐sponsored revision of the Unified Parkinson's Disease Rating Scale (MDS‐UPDRS): scale presentation and clinimetric testing results. Mov Disord 2008;23(15):2129–2170. 19025984 10.1002/mds.22340

[17] Gagnon JF , Postuma RB , Joncas S , Desjardins C , Latreille V . The Montreal Cognitive Assessment: a screening tool for mild cognitive impairment in REM sleep behavior disorder. Mov Disord 2010;25(7):936–940. 20310038 10.1002/mds.23079

[18] Mahlknecht P , Pechlaner R , Boesveldt S , Volc D , Pinter B , Reiter E , et al. Optimizing odor identification testing as quick and accurate diagnostic tool for Parkinson's disease. Mov Disord 2016;31(9):1408–1413. 27159493 10.1002/mds.26637 PMC5026160

[19] Shulman LM , Gruber‐Baldini AL , Anderson KE , Fishman PS , Reich SG , Weiner WJ . The clinically important difference on the Unified Parkinson's Disease Rating ScaleCIDs on the UPDRS. NEUR 2010;67(1):64–70. 10.1001/archneurol.2009.295 20065131

[20] Cooley SA , Heaps JM , Bolzenius JD , Salminen LE , Baker LM , Scott SE , et al. Longitudinal change in performance on the Montreal Cognitive Assessment in older adults. Clin Neuropsychol 2015;29(6):824–835. 26373627 10.1080/13854046.2015.1087596 PMC4644436

[21] Iranzo A , Serradell M , Vilaseca I , Valldeoriola F , Salamero M , Molina C , et al. Longitudinal assessment of olfactory function in idiopathic REM sleep behavior disorder. Parkinsonism Relat Disord 2013;19(6):600–604. 23529022 10.1016/j.parkreldis.2013.02.009

[22] Tang CC , Poston KL , Eckert T , Feigin A , Frucht S , Gudesblatt M , et al. Differential diagnosis of parkinsonism: a metabolic imaging study using pattern analysis. Lancet Neurol 2010;9(2):149–158. 20061183 10.1016/S1474-4422(10)70002-8 PMC4617666

[23] Iranzo A , Valldeoriola F , Lomena F , Molinuevo JL , Serradell M , Salamero M , et al. Serial dopamine transporter imaging of nigrostriatal function in patients with idiopathic rapid‐eye‐movement sleep behaviour disorder: a prospective study. Lancet Neurol 2011;10(9):797–805. 21802993 10.1016/S1474-4422(11)70152-1

[24] Iranzo A , Santamaria J , Valldeoriola F , Serradell M , Salamero M , Gaig C , et al. Dopamine transporter imaging deficit predicts early transition to synucleinopathy in idiopathic rapid eye movement sleep behavior disorder. Ann Neurol 2017;82(3):419–428. 28833467 10.1002/ana.25026

[25] van der Zande JJ , Booij J , Scheltens P , Raijmakers PG , Lemstra AW . [(123)]FP‐CIT SPECT scans initially rated as normal became abnormal over time in patients with probable dementia with Lewy bodies. Eur J Nucl Med Mol Imaging 2016;43(6):1060–1066. 26830298 10.1007/s00259-016-3312-x PMC4844648

