# Supplementary material for: Four‐Year Follow‐up of [18F]Fluorodeoxyglucose Positron Emission Tomography–Based Parkinson's Disease–Related Pattern Expression in 20 Patients with Isolated Rapid Eye Movement Sleep Behavior Disorder Shows Prodromal Progression
Source: Mov Disord. 2020 Sep 10;36(1):230–5. doi: 10.1002/mds.28260 (PMC7891341; doi:10.1002/mds.28260)
Supplement: Supplementary file 1 — Appendix S1. Supporting Information [file MDS-36-230-s001.pdf]

Supplementary Material

PDRP identification

Using the same procedures described in the Materials and Methods section, a cohort of 16 HCs (7M/9F, age 63.4±4.9 years old) and 14 PDs (9M/5F, age 65.8±9.5 years old) underwent [^{18}F]FDG-PET imaging at the UMCG. At least four of the PD patients had confirmed iRBD. At least one study has shown that PDRP expression does not differ significantly between PD patients with and without RBD.[16] PD patients were confirmed to be free of cognitive impairments for at least three years following the [^{18}F]FDG-PET scan.

A PDRP based on these scans was subsequently defined using in-house code based on a previously-defined method.[28] In this case, the PDRP was defined as the linear combinations of principal components which best distinguished between the HC and PD groups – in this case, principal component 1 (accounting for 21% of the variance in the data), component 4 (4.7% of the variance), and component 5 (4.3% of the variance).

In Supplementary Figure 1a, all voxel weights in the PDRP were overlaid on a T1 MRI template in Montreal Neurological Institute (MNI) space for visualization. All voxels in the PDRP are used for subject score calculation.

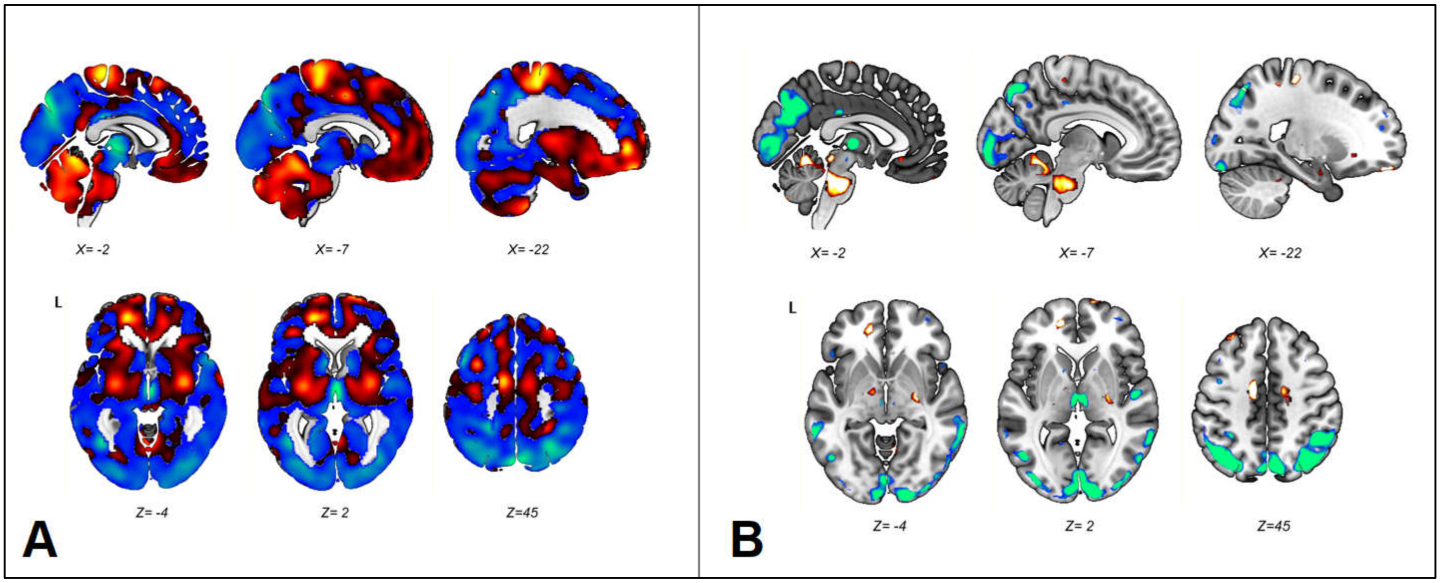

Supplementary Figure 1A. Unthresholded voxels in the PDRP. **1b.** Display of the most stable voxels in the PDRP, determined after bootstrap resampling (90% confidence interval not straddling zero).
This topography is congruent with existing literature.
Voxel values are overlaid on a T1 MRI template. Red-orange indicates positive voxel weights (relative hypermetabolism as compared to healthy controls), and blue-green indicates negative voxel weights (relative hypometabolism).
L=left. Coordinates in axial (Z) and sagittal (X) planes are in Montreal Neurological Institute (MNI) standard space.

Hoffman 3D Brain Phantom Score Correction

H3DBP images from both scanners, reconstructed and preprocessed in the same way as the human subject scans described in the Methods section (using a modified [^{18}F]FDG-PET template which excluded skull and soft tissue outside the brain[15]), were compared for PDRP expression. As the mCT40 scanner was found to have a slightly lower raw PDRP score than the mCT64, the difference (+86.17 points) was added to the raw scores of all subjects scanned on the mCT40 scanner before z-scoring to HCs (all of whom were scanned on the mCT64 scanner). This resulted in a +0.67 point PDRP z-score increase in these subjects.

Age effect on PDRP expression in HCs

Age in the 32 HCs used for PDRP derivation and z-transformation did not correlate significantly with PDRP expression, although a trend was observed (p=0.074).

In this case, the raw LOOCV scores of the 16 HCs from the PDRP derivation cohort were used for analysis, as well as the raw PDRP expression scores of the 16 HCs from the z-transformation cohort (as opposed to using z-score values).

Other

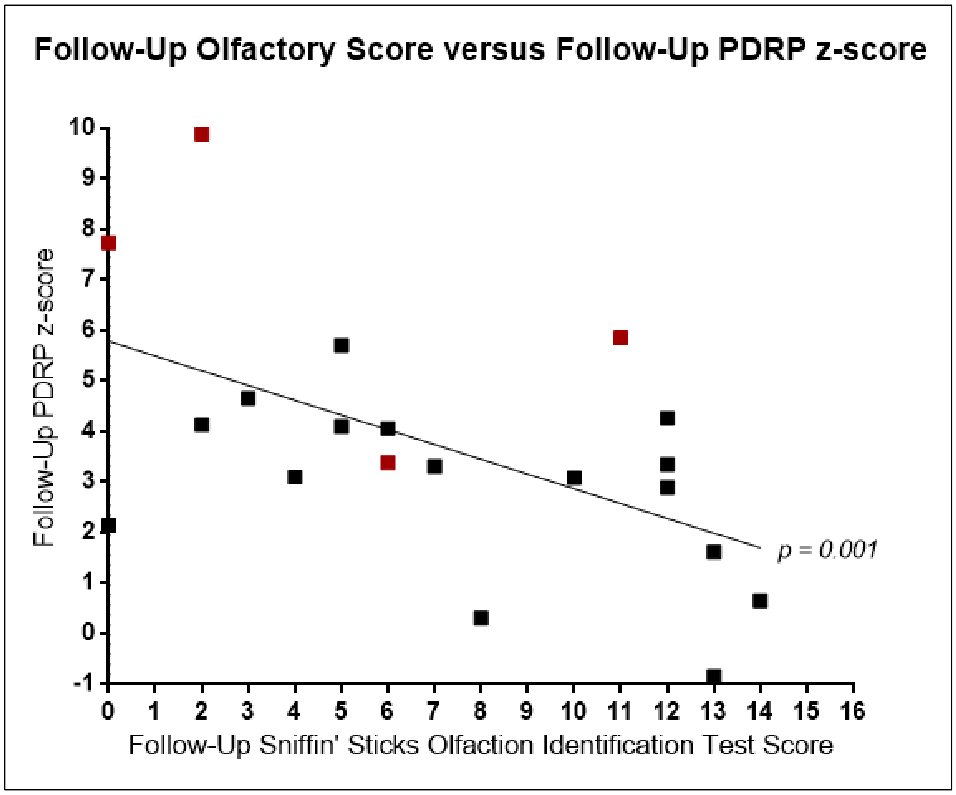

Supplementary Figure 2. Follow-up olfactory scores correlated significantly to follow-up PDRP z-scores. The four phenoconverted subjects are represented by burgundy squares.

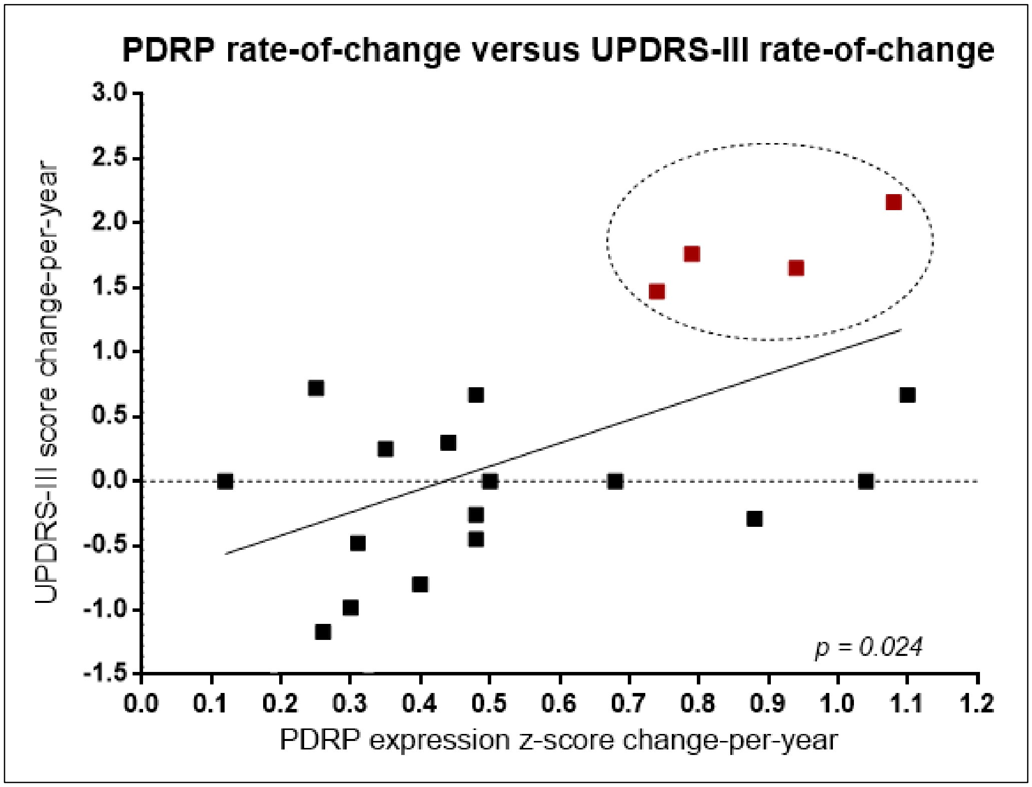

Supplementary Figure 3. PDRP expression z-score rate-of-change as compared to UPDRS motor score rate-of-change. Phenoconverted subjects (in burgundy, within the dotted oval) can be seen to have the highest rate-of-change in motor scores, as well as some of the highest rate-of-change in PDRP expression z-scores.
